# *Camellia oleifera* Seed Cake Extract Ameliorates Hyperuricemia and Renal Injury by Modulating Uric Acid Metabolism, Nrf2/Keap1 Pathway, and NLRP3 Inflammasome in Mice

**DOI:** 10.1002/fsn3.71906

**Published:** 2026-05-18

**Authors:** Liting Mai, Youliang Xie, Yingzhong Zhang, Chen Ni, Ying Xu, Lieqiang Xu, Guoshu Lin, Yinsi Lin, Ziren Su, Jing Wang, Mihong Ren

**Affiliations:** ^1^ School of Pharmaceutical Sciences Guangzhou University of Chinese Medicine Guangzhou People's Republic of China; ^2^ Guangdong Provincial Key Laboratory of Silviculture, Protection and Utilization Guangdong Academy of Forestry Guangzhou People's Republic of China; ^3^ Medical Insurance Office The Third Affiliated Hospital, Sun Yat‐Sen University, Zhaoqing Hospital Zhaoqing People's Republic of China; ^4^ Dongguan Institute of Guangzhou University of Chinese Medicine Dongguan People's Republic of China

**Keywords:** *Camellia oleifera* Abel, hyperuricemia, NLRP3 inflammasome, Nrf2/Keap1 signaling pathway, oxidative stress

## Abstract

*Camellia oleifera* seed cake is a major byproduct of the oil industry, often underutilized despite its richness in bioactive compounds. This study investigated the therapeutic effects and underlying mechanisms of *C. oleifera* seed cake extract (SCE) on potassium oxonate/hypoxanthine‐induced hyperuricemia and renal injury in mice. SCE administration significantly reduced serum uric acid levels and improved renal function parameters (creatinine and blood urea nitrogen). Mechanistically, SCE inhibited hepatic xanthine oxidase and adenosine deaminase activities and modulated renal urate transporters by downregulating URAT1 and GLUT9 while upregulating OAT1 and ABCG2, thereby inhibiting uric acid synthesis and facilitating its excretion. UHPLC–MS/MS analysis identified 134 chemical constituents in SCE, primarily phenylpropanoids and polyketides. Integrated network pharmacology predicted the involvement of inflammatory pathways, which was validated in vivo. The results confirmed that SCE alleviated renal oxidative stress and inflammation by activating the Nrf2/Keap1 signaling pathway and suppressing the NLRP3 inflammasome. These findings demonstrate that SCE exerts potent anti‐hyperuricemic and nephroprotective effects through multi‐target regulation, supporting its potential as a functional food ingredient.

## Graphical abstract

Protective mechanisms of *Camellia oleifera* seed cake extract (SCE) against hyperuricemia and renal injury via modulation of uric acid metabolism, Nrf2/Keap1 signaling, and NLRP3 inflammasome activation.

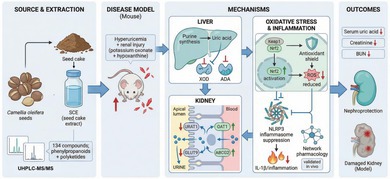

**Abbreviations**
ADA: adenosine deaminase
ASC: apoptosis‐associated speck‐like protein containing a carboxy‐terminal CARD
BUN: blood urea nitrogen
BW: body weight
Caspase1: cysteinyl aspartate specific proteinase 1
CRE: creatinine
CSK: *Camellia oleifera* seed cake
FBX: febuxostat
GCLC: glutamate‐cysteine ligase catalytic subunit
GCLM: glutamate‐cysteine ligase modifier subunit
GSH‐Px: glutathione peroxidase
HO‐1: heme oxygenase‐1
HUA: hyperuricemia
HX: hypoxanthine
Keap1: kelch‐like ECH‐associated protein 1
MDA: malondialdehyde
NLRP3: nucleotide‐binding domain, leucine‐rich‐containing family
NQO1: NAD(P)H quinone dehydrogenase 1
Nrf2: nuclear factor (erythroid‐derived 2)‐like 2
PO: potassium oxonate
ROS: reactive oxygen species
SCE: *Camellia oleifera* seed cake extract
SOD: superoxide dismutase
SUA: serum uric acid
UA: uric acid
XOD: xanthine oxidase

## 1 Introduction

An abnormal elevation of serum uric acid (SUA) defines the common metabolic condition of hyperuricemia (HUA). This pathological state develops when the synthesis of uric acid in the liver is not matched by its clearance through the kidneys (Dalbeth et al. 2021). Beyond its established role as the primary etiology of gout, HUA has emerged as a distinct contributor to a spectrum of comorbidities, including cardiovascular pathologies, hypertension, metabolic syndrome, and chronic renal failure (Zhang et al. 2019; Borghi et al. 2020). Driven by shifts in modern dietary habits and lifestyle patterns, the incidence of this disorder is escalating rapidly worldwide (Reginato et al. 2012). For instance, epidemiological data indicate that the prevalence of HUA in the Chinese population has reached approximately 18.4%, affecting younger demographics significantly (Li et al. 2021). The current therapeutic landscape for HUA is dominated by synthetic urate‐lowering therapies (ULTs), including febuxostat, allopurinol, and benzbromarone. Among these, febuxostat is a potent, selective non‐purine xanthine oxidase inhibitor that has been widely adopted as a reference drug in preclinical hyperuricemia research owing to its high selectivity and established efficacy (Saito et al. 2024). Despite their efficacy, the clinical utility of these agents is frequently compromised by significant safety concerns, ranging from severe hypersensitivity reactions to liver and kidney toxicity (Fan et al. 2021). This unfavorable risk–benefit profile has spurred an urgent search for novel, safer dietary interventions and bioactive compounds derived from functional foods capable of managing HUA and protecting against uric acid‐induced nephropathy.

*Camellia oleifera* Abel., a distinctive woody oil plant indigenous to China, has been cultivated for over 2300 years, predominantly in Hunan and Jiangxi provinces (Wu et al. 2021). While *Camellia* oil is widely celebrated as a high‐quality edible oil, the production process generates a massive amount of residue known as *C. oleifera* seed cake (CSK) (Baek et al. 2025). The annual output of CSK is estimated to exceed 800,000 tons. Unfortunately, despite being rich in bioactive compounds, CSK is currently underutilized, often discarded as waste, used as low‐value animal feed, or burned, leading to resource wastage and environmental pollution (Qiu et al. 2024). Therefore, the high‐value utilization of CSK not only aligns with the principles of sustainable agriculture but also offers a potential source of novel functional food ingredients (Zhang and Li 2018; Jiang et al. 2025). Previous studies have suggested that extracts from Camellia seeds possess antioxidant and anti‐inflammatory properties (Baek et al. 2025; Jin and Ning 2012), but the specific material basis and the molecular mechanisms underlying their potential anti‐hyperuricemic effects remain largely unexplored.

HUA‐related renal pathology arises from complex mechanisms. Beyond abnormal function of urate transporters (URAT1, GLUT9, and OAT1) that regulate uric acid homeostasis, redox imbalance and inflammatory signaling are central contributors to kidney damage (Bortolotti et al. 2021; Maiuolo et al. 2016). Uric acid excess induces ROS, which subsequently engages the NLRP3 inflammasome, driving cytokine production and hastening renal injury (Yu et al. 2021). Targeting Nrf2–Keap1‐mediated antioxidant defenses alongside NLRP3 blockade offers a compelling treatment avenue.

In a PO/HX‐induced mouse model, we comprehensively evaluated the anti‐hyperuricemic and nephroprotective efficacy of *C. oleifera* seed cake extract (SCE). Unlike prior work limited to crude polysaccharides, UHPLC–MS/MS fingerprinting defined SCE's composition, and network‐pharmacology predictions were verified in vivo. Our data newly show that SCE lowers urate and protects the kidney by regulating renal urate transporters, activating Nrf2/Keap1, and suppressing NLRP3, highlighting SCE as a sustainable functional ingredient for hyperuricemia control.

## 2 Materials and Methods

### 2.1 Reagents

The seed cake was washed, dried, and pulverized to powder of 60 mesh. The powders were extracted with hot water (60°C) in a solid–liquid ratio of 1:30 (*w*/*v*) for 60 min. After being filtered and centrifuged, threefold of ethanol (*v*) was added and the precipitation was collected to obtain SCE. SCE was concentrated, freeze‐dried, and stored at −80°C until use. SCE was provided by Guangdong Academy of Forestry Sciences. Potassium oxonate (PO, Lot: #STBH8632) and hypoxanthine (HX, Lot: #SLBZ9513) were provided by Sigma–Aldrich (St. Louis, MO, USA). The commercial kits used for detecting the levels of UA (Lot: 20200803), CRE (Lot: 20200803), BUN (Lot: 20200803), adenosine deaminase (ADA) (Lot: 20200814), XOD (Lot: 20200814), malondialdehyde (MDA, Lot: 20200805), glutathione peroxidase (GSH‐Px, Lot: 20200805), and superoxide dismutase (SOD, Lot: 20200805) were obtained by Nanjing Jiancheng Bioengineering Institute (Nanjing, China). The primary antibodies of NLRP3 (Lot: #810291), ASC (Lot: #10W2797), Caspase1 (Lot: #65K9743), Keap1 (Lot: #85d1712), and Nrf2 (Lot: #65m9929) were purchased from Affinity Biosciences (Jiangsu, China). Enzyme‐linked immunosorbent assay kits for TNF‐α (Lot: 09/2020), IL‐1β (Lot: 09/2020), and IL‐6 (Lot: 09/2020) were obtained from Shanghai MLBIO Biotechnology Co. Ltd. (Shanghai, China). RT‐qPCR primers were synthesized by Shanghai Sangon Biotech Co. Ltd. (Shanghai, China).

### 2.2 Animals and Experimental Procedures

All animal experiments were approved by the Animal Ethics Committee of Guangzhou University of Chinese Medicine (Permit ID: 20200905001) prior to the commencement of any procedures and were performed in strict accordance with the National Institutes of Health Guide for the Care and Use of Laboratory Animals (8th edition, 2011). This study was designed, conducted, and reported following the ARRIVE 2.0 guidelines.

Only male mice were used in this study for the following reasons: (1) estrogen exerts well‐documented uricosuric effects that promote renal uric acid excretion, making female mice more resistant to experimental hyperuricemia and introducing hormonal variability associated with the estrous cycle (Takiue et al. 2011); (2) the vast majority of published PO/HX‐induced hyperuricemia studies employ male mice to ensure consistency and comparability (Zhang et al. 2025); and (3) the prevalence of hyperuricemia is epidemiologically higher in males (Ngandeu‐Singwe et al. 2026).

Male Kunming mice (18–22 g, 6–8 weeks old; License: No. 44005800011756) were obtained from the Experimental Animal Center of Guangzhou University of Chinese Medicine (Guangzhou, China) and were housed under standard controlled conditions (21°C–23°C; 40%–60% humidity; 12:12 light/dark cycle) with ad libitum access to standard rodent chow and purified water. Animals were acclimatized for 7 days before the start of experiments. Acclimated mice were randomly allocated using a random number table into six groups (*n* = 6 per group) as follows (Table 1):

**TABLE 1:** Experimental group design.

Group	Model induction	Treatment (oral gavage)
(1) Normal control	0.9% saline (i.p.) + 0.5% CMC‐Na (p.o.)	0.5% CMC‐Na
(2) Model (HUA)	PO 300 mg/kg BW (i.p.) + HX 300 mg/kg BW (p.o.)	0.5% CMC‐Na
(3) FBX (positive control)	PO 300 mg/kg BW (i.p.) + HX 300 mg/kg BW (p.o.)	FBX 5 mg/kg BW
(4) SCE‐low	PO 300 mg/kg BW (i.p.) + HX 300 mg/kg BW (p.o.)	SCE 100 mg/kg BW
(5) SCE‐medium	PO 300 mg/kg BW (i.p.) + HX 300 mg/kg BW (p.o.)	SCE 200 mg/kg BW
(6) SCE‐high	PO 300 mg/kg BW (i.p.) + HX 300 mg/kg BW (p.o.)	SCE 400 mg/kg BW

The dose range of SCE was selected based on our preliminary experiments and commonly used doses of plant‐derived extracts in PO/HX‐induced hyperuricemia models. Hyperuricemia was induced by intraperitoneal injection of PO (300 mg/kg BW/day) and oral administration of HX (300 mg/kg BW/day) for 7 consecutive days. Normal mice received corresponding volumes of normal saline instead of PO/HX. One hour after model induction each day, mice in the Normal and Model groups received 0.5% CMC‐Na by oral gavage, whereas mice in the FBX and SCE groups received the corresponding treatment by oral gavage.

The sample size was determined based on our previous experience with this model and on group sizes commonly used in similar pharmacological studies, while taking animal welfare into account. At the end of the experiment, mice were anesthetized with sodium pentobarbital (50 mg/kg BW, i.p.). Blood was collected via retro‐orbital bleeding, allowed to clot for 30 min at room temperature, and centrifuged (3000 rpm, 10 min, 4°C) to isolate serum for biochemical assays. Mice were then euthanized by cervical dislocation under anesthesia. One kidney from each mouse was fixed for histological analysis; the remaining kidney tissue and liver tissue were snap‐frozen in liquid nitrogen and preserved at −80°C for ELISA, RT‐qPCR and Western blot analyses.

### 2.3 Biochemical Analysis of Serum and Liver

Serum levels of UA, CRE, BUN, SOD, GSH and MDA were measured using the assay kit. The detection method was performed by following the manufacturer's instructions. Liver tissues were homogenized in ice‐cold 100 mM Tris–HCl (PH 7.5) and centrifuged (4000 rpm, 15 min, 4°C). The supernatant fraction was used to determine XOD and ADA activities. The activities of XOD and ADA in liver were detected by commercial detection kits according to the manufacturer's protocols.

### 2.4 Histological Analysis of Kidney

Kidney tissues from mice were immobilized with 4% paraformaldehyde for 24 h and embedded in paraffin. For histopathological examination, the tissue sections (4 μm) were stained with hematoxylin–eosin (H&E) according to standard methods. Finally, renal histological lesions were appraised using an optical microscope (Olympus, Japan).

Macroscopic evaluation: Representative kidney photographs were captured for each group to assess gross morphological changes, including organ color (pallor), surface texture, and apparent swelling, compared with normal controls.

Semi‐quantitative histopathological scoring: Renal tubular injury was scored on a 0–4 scale based on the percentage of affected tubular area: 0 = no damage, 1 ≤ 25%, 2 = 25%–50%, 3 = 50%–75%, 4 ≥ 75%, as previously described (Tang et al. 2019). Pathological features assessed included tubular epithelial necrosis, tubular dilatation, cast formation, and inflammatory cell infiltration. At least five randomly selected, non‐overlapping cortical fields (×400 magnification) per section were evaluated, and the mean score per animal was calculated.

### 2.5 Determination of Renal Proinflammatory Cytokine Levels

Kidney tissues were homogenized in 10 *w*/*v* of 0.9% NaCl and centrifuged (4000 rpm, 4°C, 15 min), and supernatant protein quantified by BCA assay (Pierce, BestBio, Rockford, IL). TNF‐α, IL‐1β, and IL‐6 levels were measured by ELISA (MLBIO, China) following the manufacturer's instructions. It should be noted that these cytokines were quantified at the protein level by ELISA; RT‐qPCR was not performed for these targets.

### 2.6 Real‐Time PCR

Total RNA of kidney tissues was derived using TRIzol (Thermo Fisher Scientific, Waltham, MA, USA), followed by cDNA synthesis using the Revert Aid First Strand cDNA Synthesis Kit (Thermo Fisher Scientific, Waltham, MA, USA) to synthesize cDNA according to the manufacturer's instructions. Then, quantitative RT‐qPCR was used to analyze the cDNA using the FastStart Universal SYBR Green Master (Rox) kit (Roche, Basel, Swiss Confederation) with the following reaction conditions: 95°C for 30 s, and then 40 cycles of 95°C for 5 s and 60°C for 30 s. The relative mRNA levels of NLRP3, ASC, Caspase‐1, GCLC, GCLM, HO‐1, and NQO1 were calculated using the 2^−ΔΔCt^ method. The housekeeping gene GAPDH was employed as an internal reference to normalize the results. Primer sequences are listed in Table 2.

**TABLE 2:** Primer sequences.

Gene		Primer (5΄ to 3΄)
*NLRP3*	Forward	ATTACCCGCCCGAGAAAGG
	Reverse	TCGCAGCAAAGATCCACACAG
*ASC*	Forward	ACAATGACTGTGCTTAGAGACA
	Reverse	CACAGCTCCAGACTCTTCTTTA
*Caspase1*	Forward	AGAGGATTTCTTAACGGATGCA
	Reverse	TCACAAGACCAGGCATATTCTT
*GCLC*	Forward	AGCTCCTGGAGGAAGGCATCG
	Reverse	GGTCAGACTCGTTGGCATCATCC
*GCLM*	Forward	TGCCACCGATTTGACTGCCTTTG
	Reverse	CTGGGCTTCAATGTCAGGGATGC
*HO‐1*	Forward	ACCGCCTTCCTGCTCAACATTG
	Reverse	CCTCTGACGAAGTGACGCCATC
*NQO1*	Forward	ATCCTGCGTTTCTGTGGCTTCC
	Reverse	TCCTCCCAGACGGTTTCCAGAC
*GAPDH*	Forward	GGCAAATTCAACGGCACAGTCAAG
	Reverse	TCGCTCCTGGAAGATGGTGATGG
*GLUT9*	Forward	TGTGGACTCAATGCGATCTGGTTC
	Reverse	TGTTTCAATTCCTCCCGTGCTCAG
*URAT1*	Forward	GACCTGGACCCGATGTTCTTCTG
	Reverse	CGTGGCGTTGGACTCTGTAAGC
*OAT1*	Forward	GGAAGGTGCTGATCTTGAACTA
	Reverse	GTAGACAGTATAGTTGGGTGCA

### 2.7 Western Blot Analysis

Kidney tissues from all groups were homogenized on ice in RIPA buffer supplemented with protease inhibitor cocktail (1:100). After centrifugation (12,000 rpm, 15 min, 4°C), the supernatant was collected and total protein concentration was determined by BCA protein assay kit (BestBio, Shanghai, China). For nuclear/cytoplasmic fractionation of Nrf2, a Nuclear and Cytoplasmic Protein Extraction Kit was used according to the manufacturer's instructions. Equal amounts of protein (30–50 μg per lane) were separated by SDS‐PAGE, and transferred to PVDF membranes. After blocking with 5% non‐fat milk in TBST for 1 h at room temperature, membranes were washed three times and incubated with primary antibodies against Nrf2, Keap1, NLRP3, ASC, Caspase‐1, URAT1, GLUT9, OAT1, ABCG2, and β‐actin (all at 1:1000 dilution) overnight at 4°C, then incubated with HRP‐conjugated goat secondary antibodies (1:3000) for 1 h at room temperature. After washing with TBST three times, bands were visualized by ECL plus kit (Amersham Biosciences) and quantified with ImageJ software (NIH, USA). Band intensities were normalized to β‐actin (for total/cytoplasmic proteins) or Histone H3 (for nuclear proteins).

### 2.8 Sample Preparation and UHPLC‐Q‐Orbitrap/MS Analysis

SCE (200 μL) was extracted with methanol, ultrasonicated for 30 min, and centrifuged. The supernatant was filtered through a 0.22‐μm membrane prior to analysis. Samples were analyzed using a Vanquish Flex UHPLC coupled to a Q Exactive Orbitrap MS with an ACQUITY UPLC HSS T3 column (2.1 × 100 mm, 1.7 μm). Gradient elution used 0.1% formic acid and acetonitrile (Table 3). MS data were acquired in Full‐scan/dd‐MS2 modes with a resolution of 70,000 for Full‐scan (*m*/*z* 100–1500) and 17,500 for dd‐MS^2^ at normalized collision energies of 20, 40, and 60 eV, and processed using Progenesis QI 3.0 against the TCM Pro 2.0 database with mass error thresholds of ≤ 5 ppm. The technique acquires tandem mass spectra (MS/MS) through data‐dependent acquisition and is therefore referred to as UHPLC–MS/MS throughout this manuscript.

**TABLE 3:** Elution gradient.

Time (min)	Mobile phase	
	A (v%)	B (v%)
0	98	2
1.0	98	2
14.0	70	30
25.0	0	100
28.0	0	100
28.1	98	2
30.0	98	2

### 2.9 Target Prediction of SCE

Potential target genes of the components identified by UHPLC–MS/MS were predicted using the Swiss Target Prediction (http://www.swisstargetprediction.ch). Corresponding genes were queried via the UniProt database (https://www.uniprot.org/uploadlists/).

### 2.10 Acquisition of Public Database Targets for Hyperuricemia

Using “Hyperuricemia” as the search term, potential therapeutic targets were retrieved from GeneCards (https://www.genecards.org), Pharos (https://pharos.nih.gov/), and DISGENET (https://disgenet.com/). In addition, we re‐analyzed the GSE107821 dataset, selecting *p*adj < 0.01 for subsequent analysis.

### 2.11 Statistical Analysis

All experimental data were expressed as mean ± SEM. and analyzed with Statistical Product and Service Solutions software (SPSS 23.0; IBM Corporation Armonk, USA). The statistical differences among groups were analyzed by one‐way ANOVA with Dunnett's post hoc test and the statistical significance was set at *p* < 0.05. Graphs were generated using GraphPad Prism 8.0 software.

## 3 Results

### 3.1 SCE Ameliorated Hyperuricemia and Renal Injury

**FIGURE 1:**
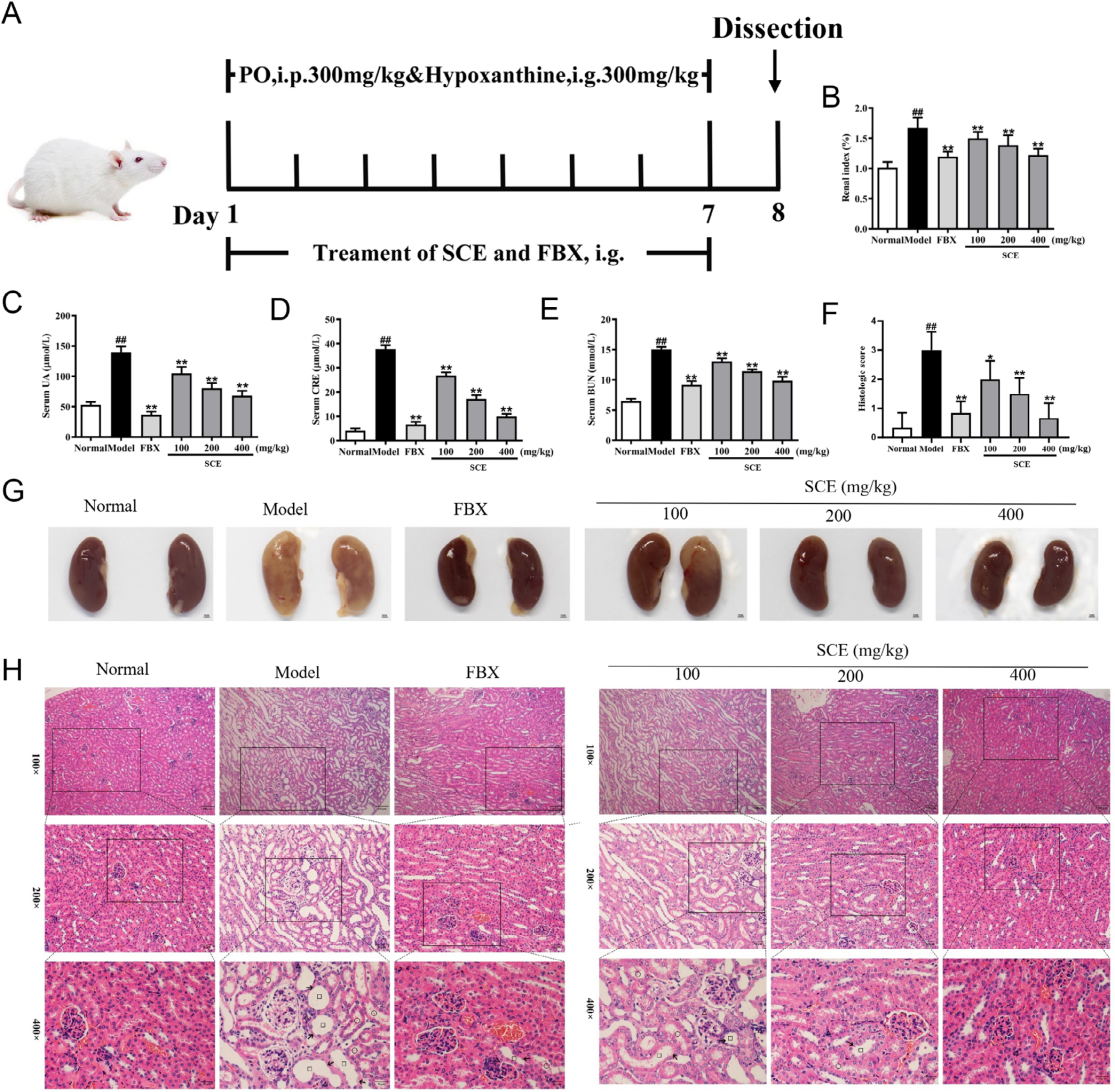
SCE ameliorated hyperuricemia and renal injury. (A) Schematic diagram of the experimental design; (B) kidney index quantified by kidney weight/body weight; serum levels of (C) UA, (D) CRE, and (E) BUN; (F) semi‐quantitative histopathological scoring of renal tubular injury; (G) representative macroscopic photographs of kidneys showing differences in color and size; (H) representative histopathological images of kidney sections (H&E staining). Black arrow represents necrotic tubular epithelial; black triangle represents inflammatory cell infiltration; black circle represents epithelial cell vacuoles; black square represents tubular ectasia. Mean ± SEM (*n* = 6). ^##^ *p* < 0.01 versus the normal group; *p < 0.05, **p < 0.01 versus the model group.

Figure 1A outlines the study design and timeline. To assess SCE's efficacy against hyperuricemia, serum uric acid was measured. As shown in Figure 1C, PO/HX elevated SUA versus normal mice (*p* < 0.01), whereas SCE at 200 or 400 mg/kg significantly lowered SUA to levels comparable to FBX (*p* < 0.01), indicating amelioration of PO/HX‐induced hyperuricemia.

Excessive SUA can result in renal uric acid deposition, fostering inflammation and injury. We examined the kidney index and renal function markers (CRE and BUN) to gauge SCE's efficacy against this damage. As illustrated in Figure 1B, the model group's kidney index was significantly elevated relative to normal mice (*p* < 0.01), an effect reversed by SCE or FBX (*p* < 0.01). Similarly, the high serum CRE and BUN levels in the model group (*p* < 0.01, Figure 1D,E) were significantly lowered following treatment with FBX or SCE (*p* < 0.01 or *p* < 0.05).

Macroscopic observation revealed that the kidneys of hyperuricemia mice appeared swollen and pale compared with normal mice, reflecting edema and possible ischemic changes, while SCE treatment restored kidney color and size to near‐normal conditions (Figure 1F). Histopathological examination further confirmed these findings. As shown in Figure 1G, PO/HX‐treated mice exhibited significant renal histopathological damage, including necrotic renal tubular epithelium, tubular dilatation, protein casts, and inflammatory cell infiltration. However, SCE‐ or FBX‐treated groups showed alleviated tubulointerstitial and glomerular lesions, with regulated proximal tubule cells and reduced protein casts. Semi‐quantitative scoring of tubular injury (Figure 1H) confirmed that the renal injury score was significantly elevated in the model group compared with the normal group (*p* < 0.01), and SCE treatment dose‐dependently reduced the injury score, with 200 and 400 mg/kg BW achieving significant improvements (*p* < 0.05 or *p* < 0.01). Collectively, these results demonstrate that SCE effectively alleviates hyperuricemia and protects against kidney injury.

### 3.2 SCE Modulates Hepatic Urate Metabolism, Redox Balance, and Inflammatory Signaling in Hyperuricemic Mice

**FIGURE 2:**
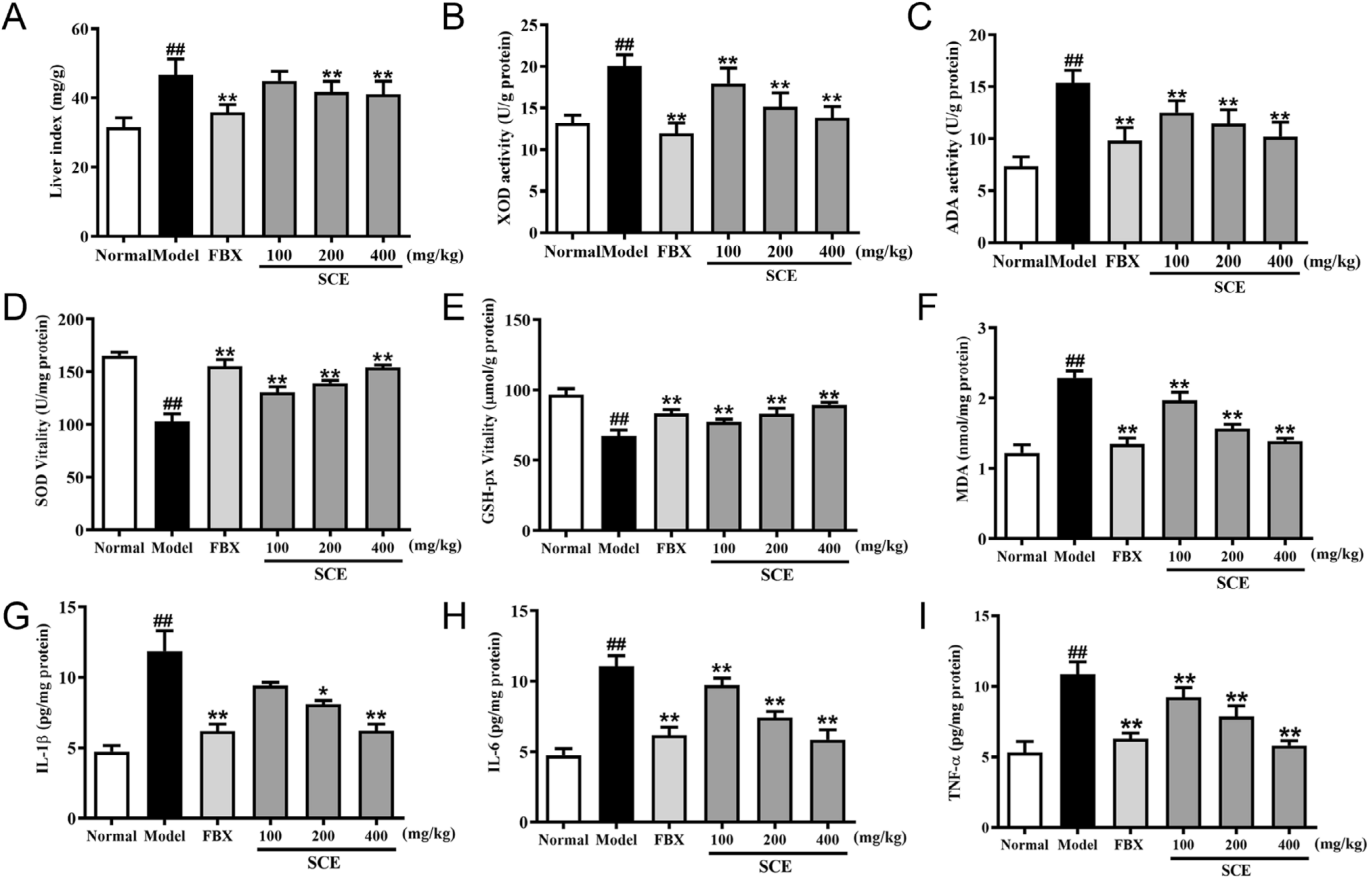
SCE modulates hepatic urate metabolism, redox balance, and inflammatory signaling in hyperuricemic mice. (A) Liver index; hepatic levels of (B) XOD and (C) ADA; renal levels of (D) SOD, (E) GSH‐Px, (F) MDA, (G) IL‐1β, (H) IL‐6, and (I) TNF‐α. Mean ± SEM (*n* = 6). ^##^ *p* < 0.01 versus the normal group; *p < 0.05, **p < 0.01 versus the model group.

To evaluate mechanisms for SCE's urate lowering, we examined hepatic XOD and ADA—key enzymes responsible for urate output. The liver index was significantly increased in the model group, while this increase was markedly reversed by treatment with FBX and SCE at doses of 200 and 400 mg/kg (Figure 2A). Hyperuricemic animals had higher XOD and ADA than normals (*p* < 0.01; Figure 2B,C), whereas SCE produced significant, dose‐dependent decreases (*p* < 0.05 or *p* < 0.01) comparable to FBX. Given the central role of ROS, we quantified renal SOD and GSH‐Px together with MDA. PO/HX significantly inhibited SOD/GSH‐Px and raised MDA (Figure 2D–F; *p* < 0.01). SCE at 100, 200, or 400 mg/kg significantly rescued enzyme activities (*p <* 0.01) and suppressed MDA (*p* < 0.01).

Hyperuricemic mice exhibited significant renal elevations of IL‐1β, IL‐6, and TNF‐α (measured by ELISA) (*p* < 0.01; Figure 2G–I). These were significantly attenuated by SCE (200, 400 mg/kg BW) and by FBX (*p* < 0.05 or *p* < 0.01). Collectively, the data suggest SCE curbs urate generation through hepatic XOD and ADA inhibition while also mitigating renal oxidative stress and inflammation.

### 3.3 SCE Modulated Renal Uric Acid Transporters in Hyperuricemia Mice

Renal urate excretion is governed by transporters that mediate reabsorption (URAT1, GLUT9) and secretion (ABCG2, OAT1). To clarify how SCE lowers circulating urate, we assessed renal expression of these transporters.

**FIGURE 3:**
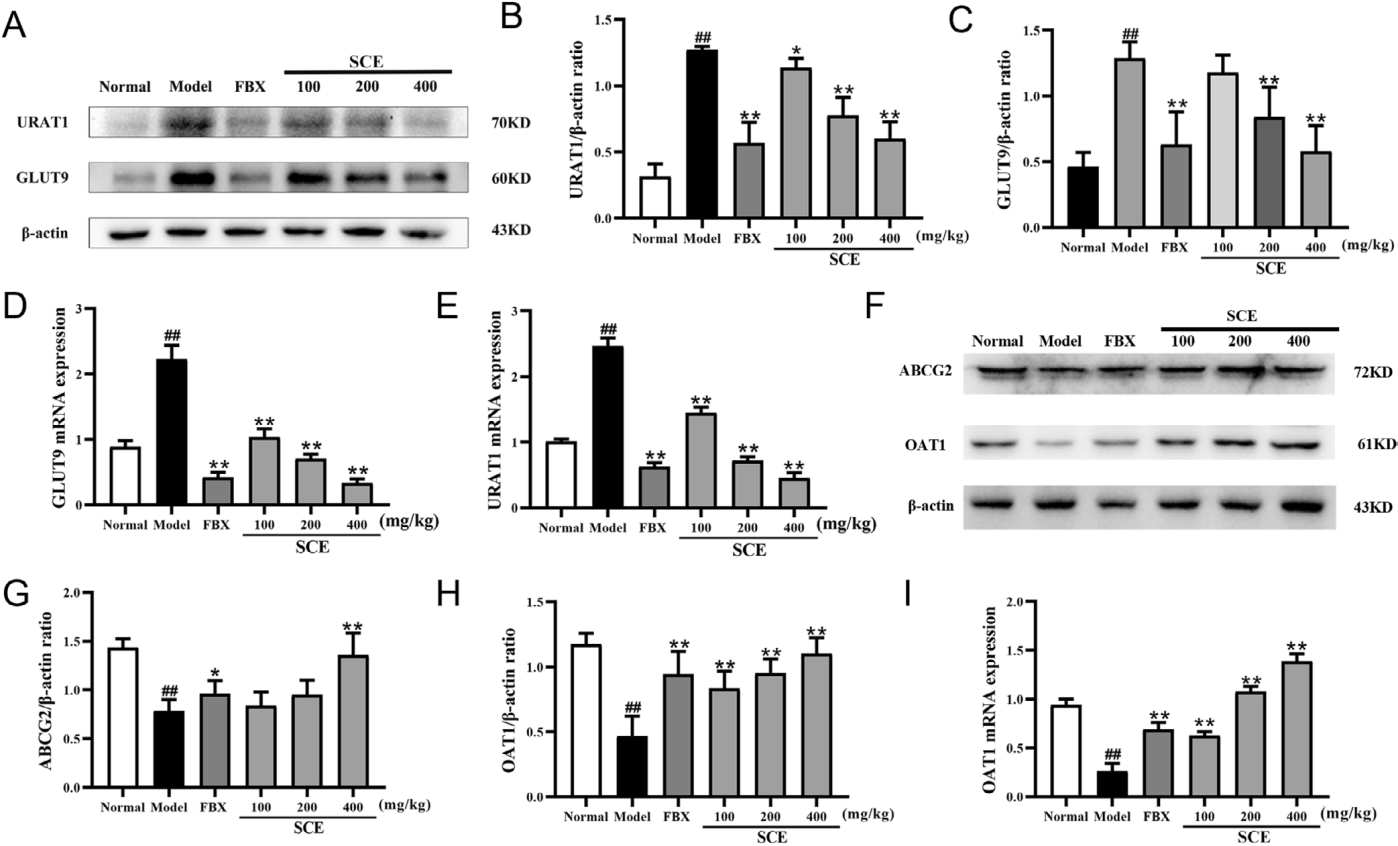
SCE modulated renal uric acid transporters in hyperuricemia mice. (A) Representative western blot images of URAT1 and GLUT9; quantification of (B) URAT1 and (C) GLUT9 protein expression; mRNA levels of (D) URAT1 and (E) GLUT9; (F) representative western blot images of ABCG2 and OAT1; quantification of (G) ABCG2 and (H) OAT1 protein expression; (I) mRNA level of OAT1. Mean ± SEM (*n* = 3–6). ^##^ *p* < 0.01 versus the normal group; *p < 0.05, **p < 0.01 versus the model group.

As shown in Figure 3A–E, PO/HX‐induced hyperuricemia markedly increased the expression of the reabsorption transporters URAT1 and GLUT9 at both the protein and mRNA levels compared with the normal group (*p <* 0.01). SCE treatment significantly downregulated URAT1 and GLUT9 expression in a dose‐dependent manner, with more pronounced effects observed at 200 and 400 mg/kg BW (*p <* 0.05 or *p <* 0.01). These findings suggest that SCE suppresses renal urate reabsorption in hyperuricemic mice.

In contrast, the protein expression of the urate secretion transporter ABCG2 and both the protein and mRNA expression of OAT1 were significantly decreased in the model group (*p <* 0.01; Figure 3F–I). Administration of SCE significantly restored ABCG2 protein expression and increased OAT1 expression at both the protein and mRNA levels (*p <* 0.05 or *p <* 0.01), indicating improved renal urate excretory capacity.

Taken together, these results demonstrate that SCE exerts a hypouricemic effect by coordinately regulating renal urate transporters, namely by downregulating the reabsorption transporters URAT1 and GLUT9 and upregulating the secretion‐associated transporters ABCG2 and OAT1.

### 3.4 SCE Activated the Nrf2/Keap1 Signaling Pathway and Its Downstream Antioxidant Genes in Hyperuricemia Mice

The Nrf2/Keap1 pathway is an endogenous antioxidant signaling cascade that plays an essential role in alleviating oxidative stress‐related diseases. To assess whether SCE activates this pathway, we analyzed the protein expression of nuclear Nrf2, cytosolic Nrf2, and Keap1 in kidney tissues by WB.

**FIGURE 4:**
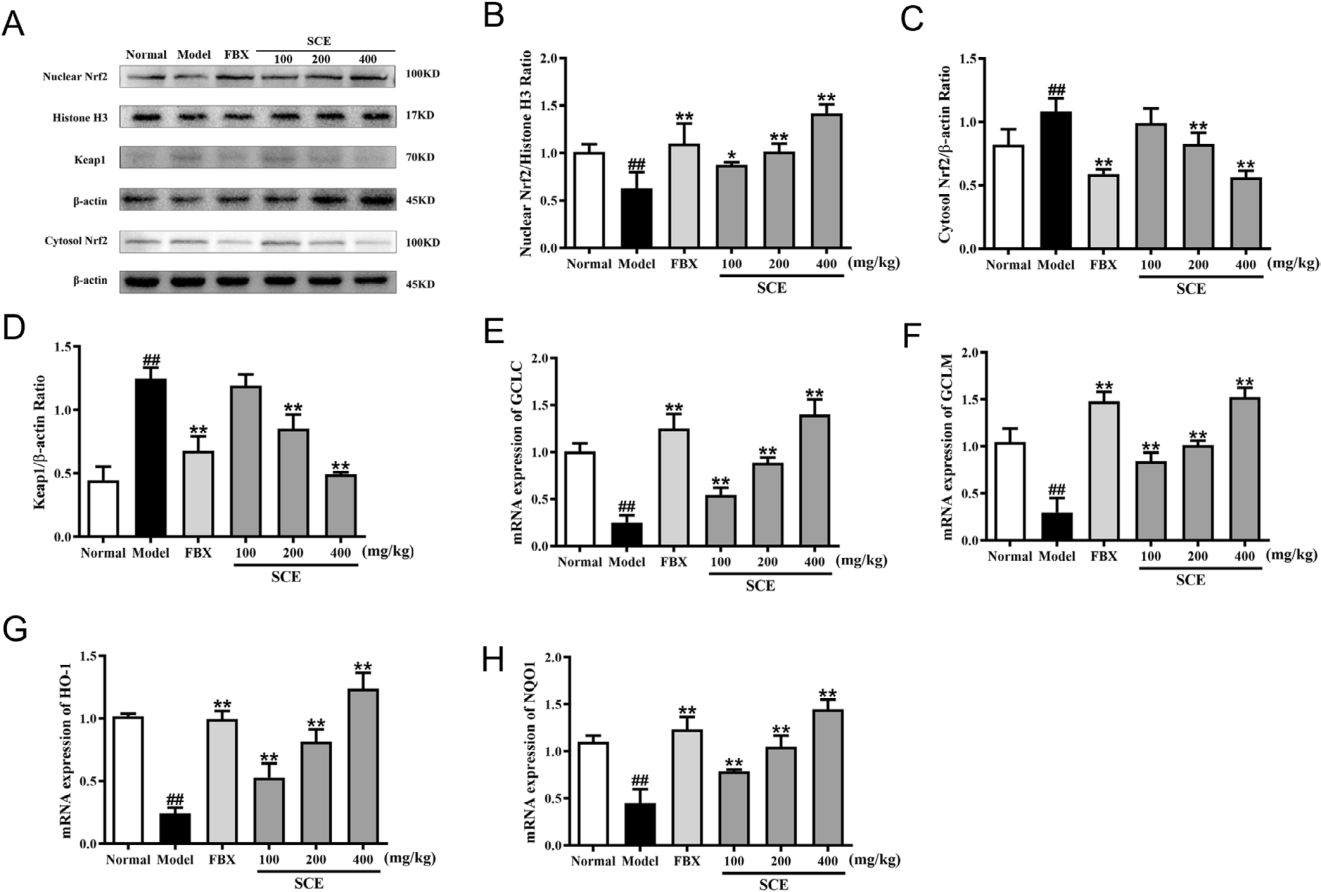
SCE activated the Nrf2/Keap1 signaling pathway and its downstream antioxidant genes in hyperuricemia mice. (A) Representative western blot images of nuclear Nrf2, cytosolic Nrf2, and Keap1; quantification of (B) nuclear Nrf2, (C) cytosolic Nrf2, and (D) Keap1 protein expression; mRNA levels of (E) GCLC, (F) GCLM, (G) HO‐1, and (H) NQO1 in kidney tissue. Mean ± SEM (*n* = 3–6). ^##^ *p* < 0.01 versus the normal group; *p < 0.05, **p < 0.01 versus the model group.

As illustrated in Figure 4A–D, the protein expression level of nuclear Nrf2 was significantly reduced, whereas cytosolic Nrf2 was increased in hyperuricemia mice (*p* < 0.01). Treatment with SCE at 200 and 400 mg/kg reversed both the downregulation of nuclear Nrf2 and the upregulation of cytosolic Nrf2 (*p* < 0.05 or *p* < 0.01). Additionally, Keap1, the inhibitory protein that sequesters Nrf2 in the cytoplasm, was upregulated by hyperuricemia (*p* < 0.01) but was restored to near‐normal levels following SCE treatment (*p* < 0.01).

To further confirm Nrf2 activation, we examined the mRNA expression of its downstream target genes. Among the genes regulated by Nrf2, GCLC and GCLM are responsible for GSH synthesis, while NQO1 and HO‐1 are antioxidant genes. As shown in Figure 4E–H, the mRNA levels of GCLC, GCLM, HO‐1, and NQO1 were remarkably lower in the model group compared with the normal group (*p* < 0.01). However, SCE or FBX administration significantly upregulated the mRNA expressions of these genes (*p* < 0.01). These observations confirm that SCE promotes the nuclear translocation and activation of Nrf2 to exert antioxidant properties.

### 3.5 Chemical Profiling of SCE by UHPLC–MS/MS Analysis

**FIGURE 5:**
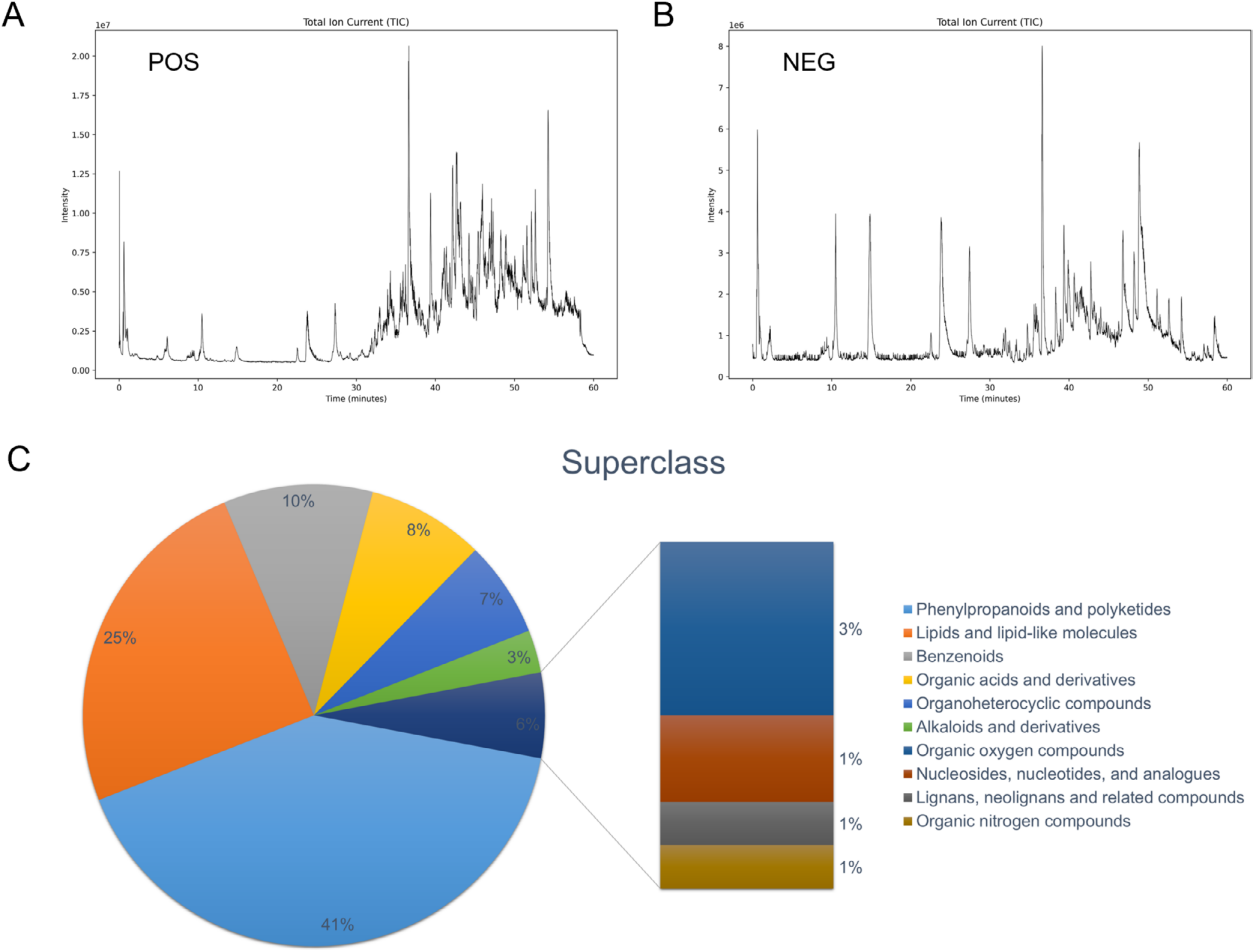
Chemical profiling of SCE by UHPLC–MS/MS analysis. (A) Total ion chromatogram (TIC) in positive ion mode; (B) total ion chromatogram (TIC) in negative ion mode; (C) pie chart showing the classification of 134 identified compounds from SCE. The compounds were classified into: phenylpropanoids and polyketides (55), lipids and lipid‐like molecules (33), benzenoids (14), organic acids and derivatives (11), organoheterocyclic compounds (9), alkaloids and derivatives (4), organic oxygen compounds (4), nucleosides, nucleotides, and analogues (2), lignans, neolignans and related compounds (1), and organic nitrogen compounds (1).

To identify the bioactive compounds in SCE, UHPLC–MS/MS analysis was performed. The total ion chromatograms (TIC) of SCE in both negative and positive ion modes are shown in Figure 5A,B. A total of 134 compounds were identified, based on accurate mass, isotope pattern, and MS/MS fragmentation matching, including 77 compounds in positive ion mode and 57 compounds in negative ion mode.

Based on structural classification, the identified compounds were grouped into ten chemical categories (Figure 5C), among which phenylpropanoids and polyketides constituted the largest proportion, followed by lipids and lipid‐like molecules, benzenoids, organic acids and derivatives, and organoheterocyclic compounds. Representative flavonoids and related polyphenols identified in SCE included kaempferol, quercetin, apigenin, naringenin, chrysin, galangin, pinocembrin, luteolin‐4′‐O‐glucoside, astragalin, isovitexin, vitexin, vicenin 2, isoschaftoside, eriocitrin, (+)‐catechin, (−)‐epicatechin, procyanidin B1, and procyanidin B2. Several kaempferol glycosides were also detected, such as kaempferol‐3‐O‐rhamnoside, kaempferol‐3‐O‐rutinoside, kaempferol 3‐O‐sophoroside, and kaempferol‐3‐O‐glucoside‐6″‐p‐coumaroyl.

In addition to flavonoids, SCE contained multiple triterpenoids and saponin‐related compounds, including betulinic acid, asiatic acid, madecassic acid, corosolic acid, maslinic acid, oleanolic acid, glycyrrhetinic acid, hederagenin, medicagenic acid, quillaic acid, soyasapogenol A, saikogenin D, and cycloastragenol. Other identified constituents included phenolic and organic acids such as gallic acid, 4‐hydroxybenzoic acid, salicylic acid, quinic acid, citric acid, fumaric acid, succinic acid, azelaic acid, sebacic acid, and suberic acid; fatty acids and related lipids such as oleic acid, linolenic acid, stearic acid, vaccenic acid, ricinoleic acid, and deoxycholic acid; and nucleosides/nucleobases such as adenine, adenosine, guanosine, guanine, hypoxanthine, and uric acid.

Notably, many of the identified constituents, especially flavonoids, procyanidins, phenolic acids, and pentacyclic triterpenoids, have previously been reported to possess xanthine oxidase‐inhibitory, antioxidant, anti‐inflammatory, and nephroprotective activities. These findings suggest that the pharmacological effects of SCE are likely associated with the synergistic action of multiple bioactive compounds rather than a single constituent. The detailed annotation information for all identified compounds is provided in Table S1.

### 3.6 Network‐Based Pharmacology Highlighted Prospective Drug Targets and Mechanistic Pathways Mediating SCE'S Anti‐Hyperuricemic Effects

**FIGURE 6:**
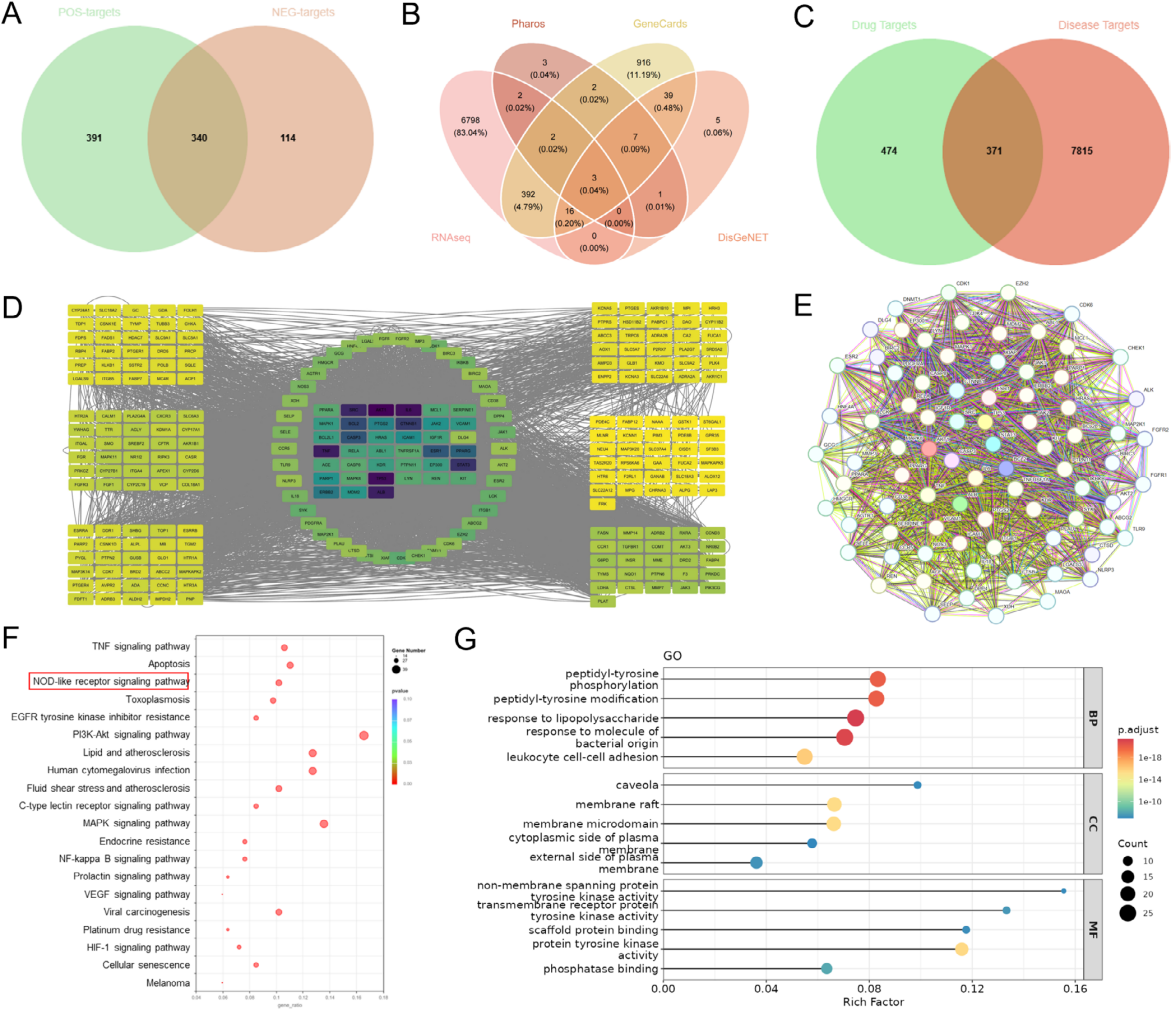
Network pharmacology analysis of SCE against hyperuricemia. (A) Drug targets predicted by Swiss Target Prediction database for compounds in positive and negative ion modes; (B) Venn diagram of disease targets obtained from Pharos, GeneCards, DisGeNET databases, and RNA‐seq data; (C) Venn diagram showing the intersection between drug targets (845) and disease targets (8186), yielding 371 common targets; (D) PPI network of 371 potential therapeutic targets constructed by Cytoscape; (E) core target PPI network (degree > 60); (F) KEGG pathway enrichment analysis of core targets; (G) GO enrichment analysis of core targets.

To systematically explore the mechanisms underlying the therapeutic effects of SCE, network pharmacology analysis was performed based on the UHPLC–MS/MS identified compounds. Drug targets were predicted using the Swiss Target Prediction database, yielding 845 potential targets for the identified compounds in both positive and negative ion modes (Figure 6A). Disease‐related targets were obtained from multiple databases, including Pharos, GeneCards, and DisGeNET, combined with RNA‐seq data, resulting in 8186 hyperuricemia‐related targets (Figure 6B).

Venn diagram analysis uncovered 371 targets common to both drug (845) and disease (8186) datasets, indicating potential SCE targets against hyperuricemia (Figure 6C). These shared targets were analyzed using Cytoscape to construct a PPI network (Figure 6D). High‐degree targets (> 60) were further filtered and visualized (Figure 6E), revealing NLRP3 as one of the most significant hub proteins.

Enrichment analysis via KEGG revealed that core targets were significantly associated with TNF signaling, apoptosis, NOD‐like receptor signaling, and PI3K‐Akt pathways (Figure 6F). GO analysis further highlighted involvement in inflammatory responses, oxidative stress regulation, and programmed cell death (Figure 6G). Collectively, these network pharmacology data suggest SCE exerts multi‐target, multi‐pathway effects against hyperuricemia, with NLRP3 inflammasome activation being a key mechanism.

### 3.7 SCE Suppressed NLRP3 Inflammasome Activation in Hyperuricemia Mice

**FIGURE 7:**
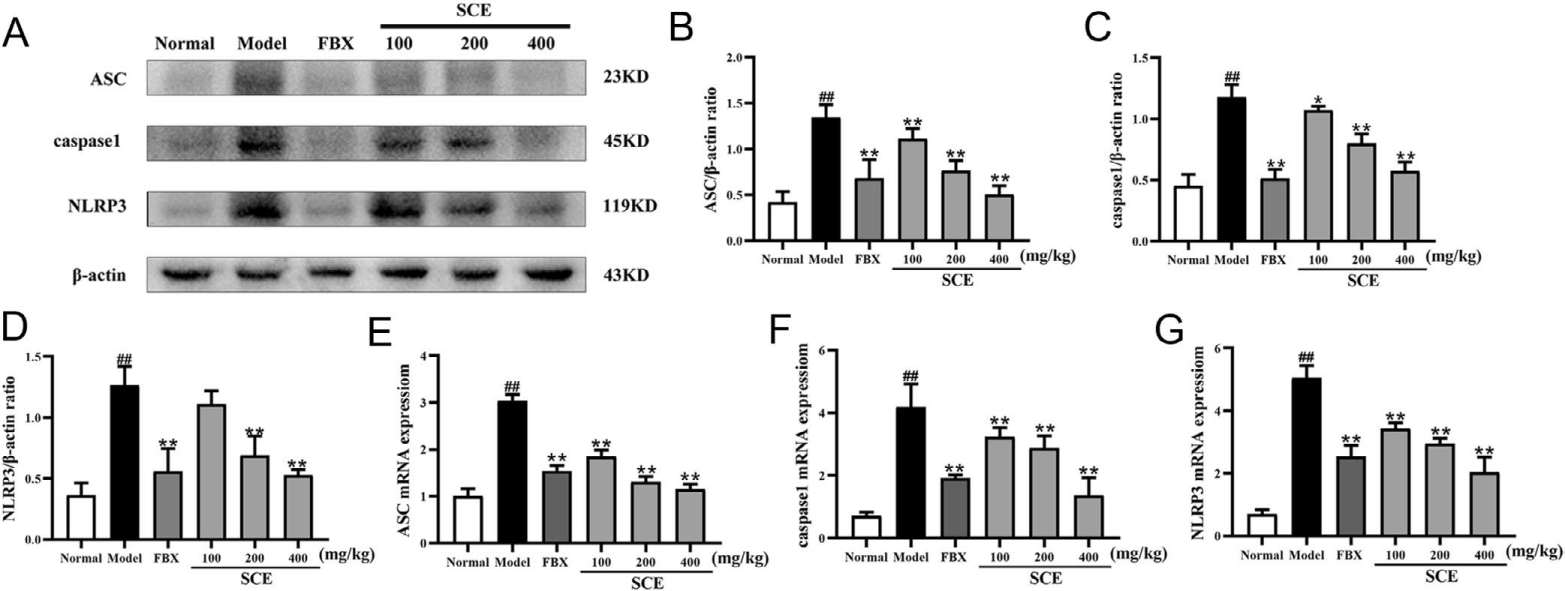
SCE suppressed NLRP3 inflammasome activation in hyperuricemia mice. (A) Representative western blot images of NLRP3, ASC, and caspase1 in kidney tissue; quantification of (B) NLRP3, (C) Caspase1, and (D) ASC protein expression by ImageJ; mRNA levels of (E) NLRP3, (F) Caspase1, and (G) ASC in kidney tissue detected by RT‐PCR. Mean ± SEM (*n* = 3–6). ^##^ *p* < 0.01 versus the normal group; *p < 0.05, **p < 0.01 versus the model group.

Based on the identification of NLRP3 as a key target through network pharmacology, we evaluated SCE's effects on NLRP3 inflammasome activation. Western blot of kidney tissues demonstrated that NLRP3, ASC, and caspase1 were significantly elevated in hyperuricemic mice relative to controls (*p* < 0.01, Figure 7A–D). Remarkably, SCE dose‐dependently attenuated the expression of these inflammasome components (*p* < 0.05 or *p* < 0.01), with the highest SCE dose showing comparable efficacy to FBX.

**FIGURE 8:**
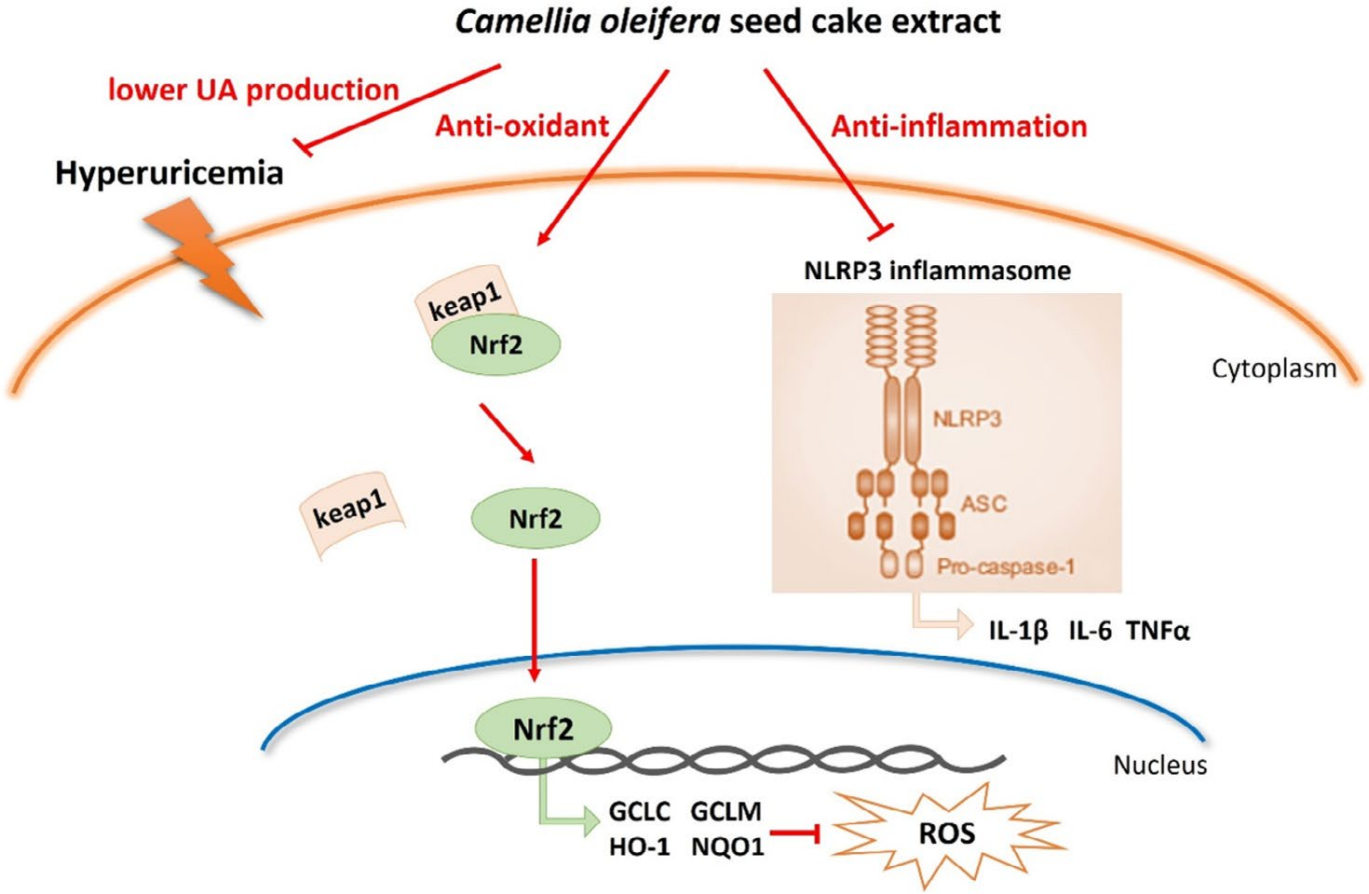
A proposed schematic diagram illustrating the protective mechanism of SCE against PO and HX‐induced hyperuricemia. Keap1, kelch‐like ECH‐associated protein 1; NLRP3 inflammasome, nucleotide‐binding domain, leucine‐rich‐containing family; Nrf2, nuclear factor (erythroid‐derived 2)‐like 2; ROS, reactive oxygen species; UA, uric acid.

Consistent with the protein expression results, the mRNA levels of NLRP3, Caspase1, and ASC were markedly elevated in hyperuricemia mice (*p* < 0.01, Figure 7E–G). SCE administration significantly suppressed the mRNA expressions of these inflammasome components in a dose‐dependent manner (*p* < 0.05 or *p* < 0.01, Figure 8).

These findings validate the network pharmacology prediction and confirm that SCE suppresses NLRP3 inflammasome activation, thereby ameliorating renal inflammation in hyperuricemia mice.

## 4 Discussion

Hyperuricemia, defined as elevated serum uric acid levels, has become an increasingly prevalent metabolic disorder associated with gout, chronic kidney disease, and cardiovascular complications (Zhang et al. 2019; Su et al. 2020). Current therapeutic agents, including xanthine oxidase inhibitors and uricosuric drugs, are clinically effective but may cause adverse effects such as hepatotoxicity and hypersensitivity reactions in some patients (Gliozzi et al. 2016). This has prompted interest in exploring natural products as alternative or complementary therapeutic options. In the present study, febuxostat was selected as the positive control drug because it is a highly selective, non‐purine xanthine oxidase inhibitor with potent urate‐lowering efficacy that is widely adopted as a reference standard in preclinical hyperuricemia research (Saito et al. 2024). Unlike allopurinol, which is a purine analog that can interfere with other steps of purine metabolism and cause hypersensitivity syndrome, febuxostat offers greater selectivity and a favorable tolerability profile in rodent models, making it an appropriate comparator for evaluating novel anti‐hyperuricemic agents (Qian et al. 2025). *C. oleifera* seed cake, a byproduct of tea oil production, has been traditionally used in Chinese medicine and contains various bioactive compounds. The present study investigated the effects of SCE on hyperuricemia and associated renal injury in PO/HX‐induced mice, and explored the underlying mechanisms.

Our results showed that SCE reduced serum uric acid levels in hyperuricemic mice in a dose‐dependent manner. Notably, SCE at 400 mg/kg BW reduced SUA to levels comparable to those achieved by febuxostat across nearly all endpoints examined, including SUA, CRE, BUN, hepatic enzyme activities, transporter expression, and inflammatory markers. The 200 mg/kg BW dose also showed significant efficacy in most parameters, while the 100 mg/kg BW dose produced more modest effects that did not reach statistical significance for some endpoints. This dose–response pattern suggests that 200 mg/kg BW approximates the minimum effective dose, while 400 mg/kg BW represents the optimal dose within the tested range. Whether doses higher than 400 mg/kg BW would confer additional benefit remains to be determined; given that the 200 and 400 mg/kg BW groups showed comparable effects on several parameters, a ceiling effect may be approaching within this dose range. Future dose‐optimization studies incorporating higher doses and pharmacokinetic analysis would help define the full dose–response curve and therapeutic window of SCE. This hypouricemic effect was accompanied by improvements in renal function parameters, including kidney index, serum creatinine, and blood urea nitrogen. Histopathological examination revealed that SCE treatment attenuated PO/HX‐induced tubular damage, including epithelial necrosis, tubular dilatation, and inflammatory cell infiltration. These findings suggest that SCE possesses both uric acid‐lowering and renoprotective properties in this experimental model.

The mechanisms underlying SCE's hypouricemic effect appear to involve regulation of both uric acid production and excretion. In the liver, SCE inhibited the activities of XOD and ADA, two enzymes involved in the purine catabolic pathway. XOD catalyzes the conversion of hypoxanthine to xanthine and subsequently to uric acid (Ullah et al. 2024), while ADA converts adenosine to inosine, providing substrates for downstream uric acid synthesis (Cristalli et al. 2001). The observed inhibition of both enzymes by SCE is consistent with reduced hepatic uric acid production, similar to the mechanism of febuxostat. Previous studies have reported that natural phenolic compounds and flavonoids can inhibit XOD activity through interaction with the enzyme's active site, and such compounds were identified in SCE through our UHPLC–MS/MS analysis.

In addition to reducing uric acid synthesis, SCE also modulated the expression of renal urate transporters involved in uric acid excretion. The kidney handles approximately 70% of daily uric acid elimination through a complex system of tubular reabsorption and secretion. URAT1 and GLUT9 are the primary transporters responsible for urate reabsorption in proximal tubules (Mishra et al. 2025), while ABCG2 and OAT1 facilitate urate secretion (Guo et al. 2023). Our results showed that hyperuricemia induced by PO/HX was associated with increased expression of URAT1 and GLUT9 and decreased expression of ABCG2 and OAT1. SCE treatment partially reversed these changes, downregulating the reabsorption transporters and upregulating the secretion transporters in a transporter‐specific manner. Specifically, SCE reduced URAT1 and GLUT9 expression at both the protein and mRNA levels, restored ABCG2 protein expression, and increased OAT1 expression at both the protein and mRNA levels. This bidirectional modulation of urate transporters would be expected to enhance net renal uric acid excretion, contributing to the observed reduction in serum uric acid levels.

The regulation of urate transporters by SCE may have clinical relevance, as genetic variants in these transporters are associated with serum uric acid levels and gout susceptibility in human populations. For instance, loss‐of‐function mutations in ABCG2 have been identified as risk factors for hyperuricemia and gout through genome‐wide association studies (Tin et al. 2019). The ability of SCE to restore ABCG2 expression in hyperuricemic mice suggests a potential mechanism for enhancing uric acid elimination. However, it should be noted that transporter expression levels do not always directly correlate with transport activity, and functional studies would be needed to confirm the impact of SCE on actual urate transport.

Oxidative stress has been implicated in the pathogenesis of hyperuricemia‐associated organ damage (Du et al. 2024). Although uric acid can act as an antioxidant in extracellular compartments, its intracellular accumulation and the XOD‐catalyzed uric acid production process can generate reactive oxygen species, contributing to oxidative injury (Du et al. 2024). Our data showed that hyperuricemic mice exhibited reduced activities of antioxidant enzymes SOD and GSH‐Px, along with elevated MDA levels, indicating increased oxidative stress in the kidney. SCE treatment restored antioxidant enzyme activities and reduced MDA levels, suggesting an antioxidant effect.

To investigate the molecular mechanism underlying SCE's antioxidant effects, we examined the Nrf2/Keap1 signaling pathway, a master regulator of cellular antioxidant responses. Under normal conditions, Nrf2 is retained in the cytoplasm by Keap1 and targeted for degradation (Sies et al. 2017). Upon oxidative stress or exposure to electrophilic compounds, Nrf2 dissociates from Keap1 and translocates to the nucleus, where it activates the transcription of antioxidant genes (Motohashi and Yamamoto 2004). Our results showed that hyperuricemia was associated with reduced nuclear Nrf2 and increased cytoplasmic Nrf2 and Keap1 levels. SCE treatment promoted nuclear Nrf2 accumulation and reduced Keap1 expression. Consistent with Nrf2 activation, the mRNA levels of downstream target genes GCLC, GCLM, HO‐1, and NQO1 were upregulated by SCE treatment. GCLC and GCLM encode subunits of glutamate‐cysteine ligase, the rate‐limiting enzyme in glutathione synthesis, while HO‐1 and NQO1 are antioxidant enzymes with cytoprotective functions (Morgenstern et al. 2024). These findings indicate that SCE can activate the Nrf2/Keap1 pathway, which may contribute to its antioxidant and renoprotective effects.

Inflammation is another important mechanism in hyperuricemia‐induced tissue damage. Elevated uric acid can activate inflammatory pathways and stimulate the production of pro‐inflammatory cytokines (Du et al. 2024). Our study showed that hyperuricemic mice had elevated renal levels of IL‐1β, IL‐6, and TNF‐α, which were reduced by SCE treatment. Network pharmacology analysis, based on the UHPLC–MS/MS‐identified compounds and hyperuricemia‐related targets, identified NLRP3 as a potential target of SCE. The NLRP3 inflammasome is a multiprotein complex that, upon activation, recruits the adaptor protein ASC and activates caspase‐1, leading to the maturation and secretion of IL‐1β and IL‐18. Uric acid crystals are known activators of the NLRP3 inflammasome.

Our experimental validation confirmed that NLRP3, ASC, and caspase‐1 were upregulated at both protein and mRNA levels in hyperuricemic mice, consistent with inflammasome activation. SCE treatment reduced the expression of these inflammasome components in a dose‐dependent manner. The suppression of NLRP3 inflammasome activation may explain, at least in part, the reduced levels of pro‐inflammatory cytokines observed in SCE‐treated mice. Of note, there is evidence suggesting crosstalk between the Nrf2 pathway and NLRP3 inflammasome, with Nrf2 activation capable of suppressing NLRP3 expression and activity (Chen et al. 2023). Therefore, SCE's effects on these two pathways may be interconnected.

The UHPLC–MS/MS analysis identified 134 compounds in SCE, with phenylpropanoids and polyketides being the most abundant class, followed by lipids and lipid‐like molecules. This chemical diversity is consistent with the multi‐target effects observed in our study. More importantly, the UHPLC‐Q‐Orbitrap MS/MS analysis revealed that SCE contains multiple flavonoids and related polyphenols, including kaempferol, quercetin, apigenin, naringenin, chrysin, galangin, pinocembrin, astragalin, vitexin, isovitexin, vicenin 2, eriocitrin, (+)‐catechin, (−)‐epicatechin, procyanidin B1, and procyanidin B2, as well as several kaempferol glycosides. Many of these compounds have been reported to exhibit xanthine oxidase‐inhibitory, antioxidant, anti‐inflammatory, and nephroprotective activities (Qian et al. 2025; Wang et al. 2023). For example, quercetin, kaempferol, apigenin, and galangin have shown xanthine oxidase inhibitory activity in previous studies, which may partly explain the reduction of hepatic XOD activity observed in the present study (Ferdiansyah et al. 2025; Yuan et al. 2025; Zhang et al. 2016). Likewise, catechins, procyanidins, and phenolic acids such as gallic acid are well known for their ROS‐scavenging capacity and may contribute to the activation of the Nrf2/Keap1 pathway and restoration of renal redox homeostasis (Xu et al. 2025; Sun et al. 2024).

In addition to flavonoids, SCE also contained multiple triterpenoids and saponin‐related compounds, including betulinic acid, asiatic acid, madecassic acid, corosolic acid, maslinic acid, oleanolic acid, glycyrrhetinic acid, hederagenin, medicagenic acid, quillaic acid, soyasapogenol A, saikogenin D, and cycloastragenol. Many of these pentacyclic triterpenoids have been reported to exert anti‐inflammatory and nephroprotective effects, including regulation of oxidative stress and suppression of NLRP3‐related inflammatory signaling (Pan et al. 2024; Sharma et al. 2018). Therefore, the anti‐hyperuricemic and renoprotective effects of SCE are likely attributable to the synergistic actions of multiple phytochemicals rather than a single component. This interpretation is also consistent with our network pharmacology results, which indicated that SCE acts through multi‐target and multi‐pathway regulation.

Several limitations of this study should be acknowledged. First, we used a chemically‐induced acute hyperuricemia model, which may not fully represent the chronic nature of hyperuricemia in humans. Studies using chronic models or genetic models of hyperuricemia would provide additional information. Second, while we identified multiple compounds in SCE through UHPLC–MS/MS, we did not determine which specific compounds are responsible for the observed effects. Bioassay‐guided fractionation studies would be needed to identify the active constituents. Third, the study was conducted in mice, and the translation of these findings to humans requires caution due to species differences in uric acid metabolism. Unlike humans, mice express uricase, which degrades uric acid to allantoin; this was addressed by using potassium oxonate, a uricase inhibitor, in our model, but differences may still exist. Fourth, only male mice were used in this study. Given the known sex‐dependent differences in uric acid metabolism mediated by estrogen, future studies should include female mice to evaluate potential sex‐specific effects. Fifth, the dose–response analysis suggests that 400 mg/kg BW is the optimal dose within the tested range, but higher doses were not evaluated; future studies should expand the dose range and include pharmacokinetic parameters to better define the therapeutic window.

In summary, our study provides evidence that *C. oleifera* seed cake extract reduces serum uric acid levels and attenuates renal injury in PO/HX‐induced hyperuricemic mice. The underlying mechanisms appear to involve inhibition of hepatic XOD and ADA activities, modulation of renal urate transporter expression, activation of the Nrf2/Keap1 antioxidant pathway, and suppression of NLRP3 inflammasome activation (Figure 8). These findings suggest that SCE may have potential as a natural product‐based intervention for hyperuricemia, though further studies are needed to identify the active compounds, confirm the mechanisms, and evaluate its efficacy and safety in clinical settings.

## 5 Conclusion

As a plant resource with both traditional medicinal and food uses, *C. oleifera* Abel. has been used for thousands of years in daily life. The present study provides experimental evidence for the application of SCE in the treatment of hyperuricemia. Our data confirm that SCE could decrease SUA levels and ameliorate renal damage in hyperuricemia mice. The hypouricemic effect of SCE was associated with inhibition of hepatic XOD and ADA activity, the activation of Nrf2/Keap1 signaling pathway, and suppression of the NLRP3 inflammasome. SCE could serve as a promising dietary therapy for hyperuricemia patients.

## Author Contributions

**Yinsi Lin:** investigation, methodology. **Ying Xu:** investigation, methodology. **Lieqiang Xu:** conceptualization, visualization. **Jing Wang:** funding acquisition, writing – review and editing, project administration, supervision. **Guoshu Lin:** software, formal analysis. **Youliang Xie:** methodology, funding acquisition, investigation. **Mihong Ren:** writing – review and editing, writing – original draft, data curation, project administration, formal analysis. **Ziren Su:** funding acquisition, project administration. **Liting Mai:** writing – original draft, conceptualization, data curation. **Chen Ni:** software, data curation. **Yingzhong Zhang:** methodology, supervision.

## Disclosure

During the preparation of this manuscript, the authors used ChatGPT (OpenAI) to assist with English language editing and improving readability. After using this tool, the authors reviewed and revised the text as needed and take full responsibility for the integrity, accuracy, and originality of the content. The AI tool was not used to generate scientific conclusions, interpret data, produce figures, or create new results.

## Ethics Statement

All animal procedures were approved by the Animal Ethics Committee of Guangzhou University of Chinese Medicine (Permit ID: 20200905001) and conducted in accordance with the National Institutes of Health Guide for the Care and Use of Laboratory Animals.

## Conflicts of Interest

The authors declare no conflicts of interest.

## Supporting information


**Table S1:** LC–MS qualitative peak annotation for SCE, including Peak ID, adduct, compound title/annotation, retention time (RT), reference *m*/*z*, precursor *m*/*z*, mass error (ppm), molecular formula, chemical superclass classification, and filtered MS/MS spectra.

## Data Availability

The data that support the findings of this study are available from the corresponding author upon reasonable request.

## References

[fsn371906-bib-0001] Baek, K.‐W. , M. T. He , W. P. Park , et al. 2025. “ *Camellia japonica* L. Seed Cake Protects Inflammatory Response in Lipopolysaccharide‐Induced Macrophages and Mice.” Journal of Food Biochemistry 2025: 8850047. 10.1155/jfbc/8850047.

[fsn371906-bib-0002] Borghi, C. , E. Agabiti‐Rosei , R. J. Johnson , et al. 2020. “Hyperuricaemia and Gout in Cardiovascular, Metabolic and Kidney Disease.” European Journal of Internal Medicine 80: 1–11. 10.1016/j.ejim.2020.07.006. 32739239

[fsn371906-bib-0003] Bortolotti, M. , L. Polito , M. G. Battelli , and A. Bolognesi . 2021. “Xanthine Oxidoreductase: One Enzyme for Multiple Physiological Tasks.” Redox Biology 41: 101882. 10.1016/j.redox.2021.101882. 33578127 PMC7879036

[fsn371906-bib-0004] Chen, Y. , X. Ye , G. Escames , et al. 2023. “The NLRP3 Inflammasome: Contributions to Inflammation‐Related Diseases.” Cellular & Molecular Biology Letters 28: 51. 10.1186/s11658-023-00462-9. 37370025 PMC10303833

[fsn371906-bib-0005] Cristalli, G. , S. Costanzi , C. Lambertucci , et al. 2001. “Adenosine Deaminase: Functional Implications and Different Classes of Inhibitors.” Medicinal Research Reviews 21: 105–128. 11223861 10.1002/1098-1128(200103)21:2<105::aid-med1002>3.0.co;2-u

[fsn371906-bib-0006] Dalbeth, N. , A. L. Gosling , A. Gaffo , and A. Abhishek . 2021. “Gout.” Lancet 397: 1843–1855. 10.1016/S0140-6736(21)00569-9. 33798500

[fsn371906-bib-0007] Du, L. , Y. Zong , H. Li , et al. 2024. “Hyperuricemia and Its Related Diseases: Mechanisms and Advances in Therapy.” Signal Transduction and Targeted Therapy 9: 212. 10.1038/s41392-024-01916-y. 39191722 PMC11350024

[fsn371906-bib-0008] Fan, M. , J. Liu , B. Zhao , et al. 2021. “Comparison of Efficacy and Safety of Urate‐Lowering Therapies for Hyperuricemic Patients With Gout: A Meta‐Analysis of Randomized, Controlled Trials.” Clinical Rheumatology 40: 683–692. 10.1007/s10067-020-05272-4. 32654080

[fsn371906-bib-0009] Ferdiansyah, M. K. , Y. H. Kim , K. P. Kim , and M. K. Kim . 2025. “Quercetin as the Primary Xanthine Oxidase Inhibitor Compound in *Maclura tricuspidata* Leaf.” Natural Product Research 39: 6504–6508. 10.1080/14786419.2024.2377317. 39004844

[fsn371906-bib-0010] Gliozzi, M. , N. Malara , S. Muscoli , and V. Mollace . 2016. “The Treatment of Hyperuricemia.” International Journal of Cardiology 213: 23–27. 10.1016/j.ijcard.2015.08.087. 26320372

[fsn371906-bib-0011] Guo, X.‐L. , Y. Y. Gao , Y. X. Yang , et al. 2023. “Amelioration Effects of α‐Viniferin on Hyperuricemia and Hyperuricemia‐Induced Kidney Injury in Mice.” Phytomedicine: International Journal of Phytotherapy and Phytopharmacology 116: 154868. 10.1016/j.phymed.2023.154868. 37209608

[fsn371906-bib-0012] Jiang, N. , Z. Xu , S. Lu , et al. 2025. Advances in Valorization of *Camellia oleifera* Abel. Seed Cake: A Review on the Bioactive Components, Health Benefits, Extraction Methods, and Potential Food Applications. Food Research International. 10.1016/j.foodres.2025.116134. 40263822

[fsn371906-bib-0013] Jin, X. , and Y. Ning . 2012. “Antioxidant and Antitumor Activities of the Polysaccharide From Seed Cake of *Camellia oleifera* Abel.” International Journal of Biological Macromolecules 51: 364–368. 10.1016/j.ijbiomac.2012.05.033. 22683896

[fsn371906-bib-0014] Li, Y. , Z. Shen , B. Zhu , H. Zhang , X. Zhang , and X. Ding . 2021. “Demographic, Regional and Temporal Trends of Hyperuricemia Epidemics in Mainland China From 2000 to 2019: A Systematic Review and Meta‐Analysis.” Global Health Action 14: 1874652. 10.1080/16549716.2021.1874652. 33475474 PMC7833047

[fsn371906-bib-0015] Maiuolo, J. , F. Oppedisano , S. Gratteri , C. Muscoli , and V. Mollace . 2016. “Regulation of Uric Acid Metabolism and Excretion.” International Journal of Cardiology 213: 8–14. 10.1016/j.ijcard.2015.08.109. 26316329

[fsn371906-bib-0016] Mishra, S. , R. K. Harwansh , and R. Mazumder . 2025. “URAT1 and GLUT9 as Drug Targets in Gout: Progress in Transporter‐Directed Therapies and Delivery Technologies.” Inflammopharmacology 33: 6535–6553. 10.1007/s10787-025-01997-3. 41065961

[fsn371906-bib-0017] Morgenstern, C. , I. Lastres‐Becker , B. C. Demirdöğen , et al. 2024. “Biomarkers of NRF2 Signalling: Current Status and Future Challenges.” Redox Biology 72: 103134. 10.1016/j.redox.2024.103134. 38643749 PMC11046063

[fsn371906-bib-0018] Motohashi, H. , and M. Yamamoto . 2004. “Nrf2‐Keap1 Defines a Physiologically Important Stress Response Mechanism.” Trends in Molecular Medicine 10: 549–557. 10.1016/j.molmed.2004.09.003. 15519281

[fsn371906-bib-0019] Ngandeu‐Singwe, M. , J. R. Nkeck , A. Hamroun , et al. 2026. “Worldwide Trends in Hyperuricaemia From 2000 to 2023: A Systematic Review and Modelling Analysis.” Lancet. Rheumatology 8: e346–e362. 10.1016/S2665-9913(25)00344-3. 41662849

[fsn371906-bib-0020] Pan, D. , Y. Qu , C. Shi , et al. 2024. “Oleanolic Acid and Its Analogues: Promising Therapeutics for Kidney Disease.” Chinese Medicine 19: 74. 10.1186/s13020-024-00934-w. 38816880 PMC11140902

[fsn371906-bib-0021] Qian, Y. , Y. Zhang , Y. Chen , et al. 2025. “Linarin Attenuates Hyperuricemic Nephropathy by Modulating Nrf2/Keap1 and TLR4/NF‐κB Signaling Pathways: Linarin Attenuates Hyperuricemic Nephropathy.” Phytomedicine: International Journal of Phytotherapy and Phytopharmacology 139: 156440. 10.1016/j.phymed.2025.156440. 39908585

[fsn371906-bib-0022] Qiu, C. , Z. Chen , F. Hu , et al. 2024. Simultaneous Extraction of Oil, Protein and Polysaccharide From Residual Seed Cake of *Camellia oleifera* Abel. Using Three Phase Partitioning. Industrial Crops and Products. 10.1016/j.indcrop.2023.117994.

[fsn371906-bib-0023] Reginato, A. M. , D. B. Mount , I. Yang , and H. K. Choi . 2012. “The Genetics of Hyperuricaemia and Gout.” Nature Reviews Rheumatology 8: 610–621. 10.1038/nrrheum.2012.144. 22945592 PMC3645862

[fsn371906-bib-0024] Saito, Y. , A. Tanaka , H. Yoshida , et al. 2024. “Effects of Xanthine Oxidase Inhibition by Febuxostat on Lipid Profiles of Patients With Hyperuricemia: Insights From Randomized PRIZE Study.” Nutrients 16: 2324. 10.3390/nu16142324. 39064767 PMC11280470

[fsn371906-bib-0025] Sharma, H. , P. Kumar , R. R. Deshmukh , A. Bishayee , and S. Kumar . 2018. “Pentacyclic Triterpenes: New Tools to Fight Metabolic Syndrome.” Phytomedicine: International Journal of Phytotherapy and Phytopharmacology 50: 166–177. 10.1016/j.phymed.2018.09.011. 30466975

[fsn371906-bib-0026] Sies, H. , C. Berndt , and D. P. Jones . 2017. “Oxidative Stress.” Annual Review of Biochemistry 86: 715–748. 10.1146/annurev-biochem-061516-045037. 28441057

[fsn371906-bib-0027] Su, H.‐Y. , C. Yang , D. Liang , and H. F. Liu . 2020. “Research Advances in the Mechanisms of Hyperuricemia‐Induced Renal Injury.” BioMed Research International 2020: 5817348. 10.1155/2020/5817348. 32685502 PMC7336201

[fsn371906-bib-0028] Sun, L. , S. Wei , C. Wang , et al. 2024. “Procyanidin Capsules Provide a New Option for Long‐Term ROS Scavenging in Chronic Inflammatory Diseases.” Materials Today Bio 29: 101310. 10.1016/j.mtbio.2024.101310. PMC11554635 39534678

[fsn371906-bib-0029] Takiue, Y. , M. Hosoyamada , M. Kimura , and H. Saito . 2011. “The Effect of Female Hormones Upon Urate Transport Systems in the Mouse Kidney.” Nucleosides, Nucleotides & Nucleic Acids 30: 113–119. 10.1080/15257770.2010.551645. 21360409

[fsn371906-bib-0030] Tang, C. , H. Han , Z. Liu , et al. 2019. “Activation of BNIP3‐Mediated Mitophagy Protects Against Renal Ischemia‐Reperfusion Injury.” Cell Death & Disease 10: 677. 10.1038/s41419-019-1899-0. 31515472 PMC6742651

[fsn371906-bib-0031] Tin, A. , J. Marten , V. L. Halperin Kuhns , et al. 2019. “Target Genes, Variants, Tissues and Transcriptional Pathways Influencing Human Serum Urate Levels.” Nature Genetics 51: 1459–1474. 10.1038/s41588-019-0504-x. 31578528 PMC6858555

[fsn371906-bib-0032] Ullah, Z. , P. Yue , G. Mao , et al. 2024. “A Comprehensive Review on Recent Xanthine Oxidase Inhibitors of Dietary Based Bioactive Substances for the Treatment of Hyperuricemia and Gout: Molecular Mechanisms and Perspective.” International Journal of Biological Macromolecules 278: 134832. 10.1016/j.ijbiomac.2024.134832. 39168219

[fsn371906-bib-0033] Wang, X. , L. Dong , Y. Dong , Z. Bao , and S. Lin . 2023. “Corn Silk Flavonoids Ameliorate Hyperuricemia via PI3K/AKT/NF‐κB Pathway.” Journal of Agricultural and Food Chemistry 71: 9429–9440. 10.1021/acs.jafc.3c03422. 37294890

[fsn371906-bib-0034] Wu, B. , C. Ruan , A. H. Shah , et al. 2021. “Identification of miRNA‐mRNA Regulatory Modules Involved in Lipid Metabolism and Seed Development in a Woody Oil Tree (*Camellia oleifera*).” Cells 11: 71. 10.3390/cells11010071. 35011633 PMC8750442

[fsn371906-bib-0035] Xu, E. , Y. Sun , Z. Yu , and J. Zheng . 2025. “Epigallocatechin Gallate Alleviates Cisplatin Induced Intestinal Injury in Rats via Inhibiting NRF2/Keap1 Signaling Pathway and Regulating Gut Microbiota and Metabolites.” Molecular Nutrition & Food Research 69: e202400784. 10.1002/mnfr.202400784. 39757492

[fsn371906-bib-0036] Yu, P. , X. Zhang , N. Liu , L. Tang , C. Peng , and X. Chen . 2021. “Pyroptosis: Mechanisms and Diseases.” Signal Transduction and Targeted Therapy 6: 128. 10.1038/s41392-021-00507-5. 33776057 PMC8005494

[fsn371906-bib-0037] Yuan, J. , D. Wu , J. Liu , et al. 2025. “Identification of Bioactive Compounds From Citri Reticulatae Pericarpium and Evaluation of Their Uric Acid‐Lowering Activity.” Plant Foods for Human Nutrition 80: 97. 10.1007/s11130-025-01337-4. 40117004

[fsn371906-bib-0038] Zhang, C. , G. Zhang , J. Pan , and D. Gong . 2016. “Galangin Competitively Inhibits Xanthine Oxidase by a Ping‐Pong Mechanism.” Foodservice Research International 89: 152–160. 10.1016/j.foodres.2016.07.021. 28460900

[fsn371906-bib-0039] Zhang, S. , K. Li , H. Zhang , et al. 2025. “Progress in Animal Models for Studying Hyperuricemia.” Frontiers in Pharmacology 16: 1636205. 10.3389/fphar.2025.1636205. 40918521 PMC12411503

[fsn371906-bib-0040] Zhang, S. , and X. Li . 2018. “Hypoglycemic Activity In Vitro of Polysaccharides From *Camellia oleifera* Abel. Seed Cake.” International Journal of Biological Macromolecules 115: 811–819. 10.1016/j.ijbiomac.2018.04.054. 29654860

[fsn371906-bib-0041] Zhang, S. , Y. Wang , J. Cheng , et al. 2019. “Hyperuricemia and Cardiovascular Disease.” Current Pharmaceutical Design 25: 700–709. 10.2174/1381612825666190408122557. 30961478

