# Correction to “*Camellia oleifera* Seed Cake Extract Ameliorates Hyperuricemia and Renal Injury by Modulating Uric Acid Metabolism, Nrf2/Keap1 Pathway, and NLRP3 Inflammasome in Mice”

**DOI:** 10.1002/fsn3.72120

**Published:** 2026-07-14

Mai, L. , Y. Xie , Y. Zhang , et al. 2026. “
*Camellia oleifera* Seed Cake Extract Ameliorates Hyperuricemia and Renal Injury by Modulating Uric Acid Metabolism, Nrf2/Keap1 Pathway, and NLRP3 Inflammasome in Mice.” Food Science & Nutrition 14, no. 5: e71906. 10.1002/fsn3.71906.42165050 PMC13184386

In the Acknowledgment section, the grant source of “the Science and Technology Planning Project of Guangdong Province (2025WDZC‐LKY02)” was missing. The updated Acknowledgment section is as follows:

This work was supported by grants from the Science and Technology Planning Project of Guangdong Province (2025WDZC‐LKY02), the Guangdong Forestry Bureau (广东省林业局) under the Guangdong Forestry Science and Technology Innovation Project (2026KJCX004, 2023KJCX003), and from the Natural Science Foundation of Guangdong Province (2021A1515010160, 2018A030313408).

We apologize for this error.

